# Life‐Threatening Acute Tumor Lysis Syndrome With Multiorgan Failure Following First Cycle of Bendamustine–Rituximab in Chronic Lymphocytic Leukemia: A Case Report and Brief Review

**DOI:** 10.1002/ccr3.72548

**Published:** 2026-04-15

**Authors:** Malegna Temesgen Garuma, Kebede H. Begna, Tesfaye Belete Amare, Mengisteab Kassahun Desta, Derebe Shashigo Leilago, Humud Mohammed Ahmed

**Affiliations:** ^1^ Department of Internal Medicine St. Paul's Hospital Millennium Medical College (SPHMMC) Addis Ababa Ethiopia; ^2^ Hematology‐Oncology Unit, Department of Internal Medicine SPHMMC Addis Ababa Ethiopia; ^3^ Department of Internal Medicine, Division of Hematology Mayo Clinic Rochester Minnesota USA

**Keywords:** acute kidney injury, acute tumor lysis syndrome, Bendamustine–rituximab, chronic lymphocytic leukemia, hematologic emergency, multiorgan failure

## Abstract

Chronic lymphocytic leukemia is not uniformly low risk for tumor lysis syndrome. This case shows that first‐cycle bendamustine–rituximab in patients with high disease burden can precipitate severe TLS with acute kidney injury and multiorgan failure. Individualized prophylaxis, patient education, and close monitoring during early therapy are essential.

## Graphical abstract

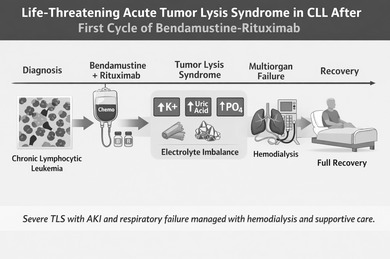

**Abbreviations**
BP: Blood Pressure
BTK: Bruton Tyrosine Kinase
CLL: Chronic Lymphocytic Leukemia
ECG: Electrocardiogram
GCS: Glasgow Coma Scale
LDH: Lactate Dehydrogenase
TLS: Tumor Lysis Syndrome
WBC: White Blood Cell count

## 1 Introduction

Chronic lymphocytic leukemia (CLL) is the most common adult leukemia worldwide and is characterized by clonal proliferation and accumulation of mature, immunologically dysfunctional B lymphocytes. The disease course is heterogeneous, ranging from indolent to aggressive. Risk stratification, such as Rai and Binet systems, guides management [1].

Tumor lysis syndrome (TLS) is a potentially fatal metabolic complication caused by the rapid destruction of malignant cells. This leads to the release of intracellular content, causing hyperuricemia, hyperkalemia, hyperphosphatemia, and hypocalcemia. Severe TLS can cause acute kidney injury, arrhythmias, seizures, and death [2]. TLS is classically associated with high‐grade lymphomas and acute leukemias but is considered uncommon in CLL because of its typically low proliferative rate [3].

Emerging clinical experience and case reports increasingly recognize that CLL patients presenting with advanced disease features including massive leukocytosis, bulky lymphadenopathy, marked organomegaly, and elevated baseline uric acid or lactate dehydrogenase (LDH) levels represent a distinct high‐risk population for TLS, particularly when exposed to potent cytoreductive therapies [1, 2]. Bendamustine–rituximab is widely used, especially in resource‐limited settings where first‐line Bruton tyrosine kinase (BTK) inhibitors may be unavailable or contraindicated [4, 5]. Although generally well tolerated, the regimen's rapid cytoreductive effects can, in rare but critical circumstances, precipitate life‐threatening TLS [3, 6]. Severe TLS requiring urgent hemodialysis in CLL patients treated with bendamustine–rituximab remains infrequently reported, particularly in resource‐constrained contexts, highlighting the clinical and educational significance of the present case [2, 4].

## 2 Case History/Examination

### 2.1 Baseline (Day 0): Initial Diagnosis and Pretreatment Assessment

A previously healthy 54‐year‐old man presented to our institution 3 months before the index presentation with a history of progressive constitutional symptoms, including profound fatigue, marked anorexia, and significant unintentional weight loss. On detailed history, the patient reported gradually progressive abdominal distension and progressive left‐sided dragging discomfort. Physical examination at the time of initial diagnosis revealed conjunctival pallor consistent with anemia, multiple bulky nontender lymph nodes in bilateral cervical, axillary, and inguinal chains, and marked splenomegaly extending approximately 20 cm below the left costal margin on palpation. The systemic examination was otherwise unremarkable, with no evidence of hepatomegaly or other organ involvement.

Baseline hematological investigations revealed marked leukocytosis with a white blood cell count of 300 × 10^3^/μL, hemoglobin of 10 g/dL, and platelet count of 110 × 10^3^/μL. Renal function and electrolytes at baseline were reassuringly normal or near normal, with serum creatinine of 0.8 mg/dL (normal 0.51–0.95 mg/dL), uric acid of 6.4 mg/dL (normal 2.6–6.0 mg/dL), and potassium of 4.0 mmol/L (normal 3.5–5.1 mmol/L). Peripheral blood smear and flow cytometry analysis confirmed a clonal B‐cell population with aberrant CD5 and CD23 coexpression, lambda light‐chain restriction, and negativity for CD10, FMC7, and CD103, consistent with CLL/small lymphocytic lymphoma (summary presented in Table 2). Lymph node biopsy and additional imaging studies supported the diagnosis of advanced CLL. The patient was formally staged as Rai stage III and Binet stage B, reflecting advanced disease with anemia, lymphocytosis, and organomegaly. Despite the advanced stage, the clinical team classified the patient as having standard‐risk disease suitable for observation or sequential therapy, failing to recognize the high‐risk features inherent to this particular presentation.

### 2.2 Day 0–1: Treatment Initiation Without Prophylaxis

On the day of initial presentation to the hematology‐oncology service, after confirmation of CLL diagnosis and staging, first‐cycle bendamustine–rituximab chemotherapy was initiated. The standard dose of intravenous bendamustine was administered on Days 1 and 2, and rituximab infusion was begun. At the time of treatment initiation, the patient received routine counseling regarding general treatment‐related complications and potential chemotherapy adverse effects. Notably, no TLS prophylaxis, including allopurinol or rasburicase, was prescribed. This likely reflected underestimation of the patient's TLS risk, given the common perception that CLL is generally low risk for TLS. The patient was discharged home with instructions to maintain adequate oral hydration and to return immediately for any concerning symptoms.

### 2.3 Day 3: Acute Clinical Deterioration and Hospital Readmission

Seventy‐two hours following the initiation of bendamustine–rituximab, the patient returned to the emergency department in acute distress. He reported rapid clinical deterioration with progressive worsening of fatigue, marked poor oral intake with inability to tolerate food or liquids, repeated episodes of vomiting, passage of dark‐colored urine, and progressive bilateral leg swelling. On examination, he appeared acutely ill and in obvious respiratory distress, requiring supplemental oxygen therapy at 10 L/min via a nonrebreather mask to maintain adequate oxygenation. Vital signs revealed blood pressure 120/70 mmHg, heart rate 120 beats per minute, respiratory rate 28 breaths per minute, temperature 36.8°C, and oxygen saturation 90% on oxygen. Glasgow Coma Scale was 15/15. Physical examination of the chest revealed bilateral diffuse coarse crepitations consistent with pulmonary edema. Grade 2 bilateral pitting pedal edema was evident. The patient was immediately admitted to the intensive care unit with a presumptive diagnosis of TLS‐related acute kidney injury and multiorgan failure.

## 3 Differential Diagnosis, Investigations, and Treatment

Given the acute presentation, a broad differential diagnosis was initially entertained, including sepsis with acute kidney injury, direct chemotherapy‐induced nephrotoxicity unrelated to TLS, acute leukemic transformation, and acute decompensated heart failure with secondary renal impairment. However, the temporal relationship between chemotherapy initiation and acute clinical decompensation, combined with the characteristic and severe laboratory abnormalities, strongly supported the diagnosis of acute TLS with secondary complications.

### 3.1 Urgent Laboratory Investigations on Day 3 Revealed Catastrophic Metabolic Derangements

**FIGURE 1:**
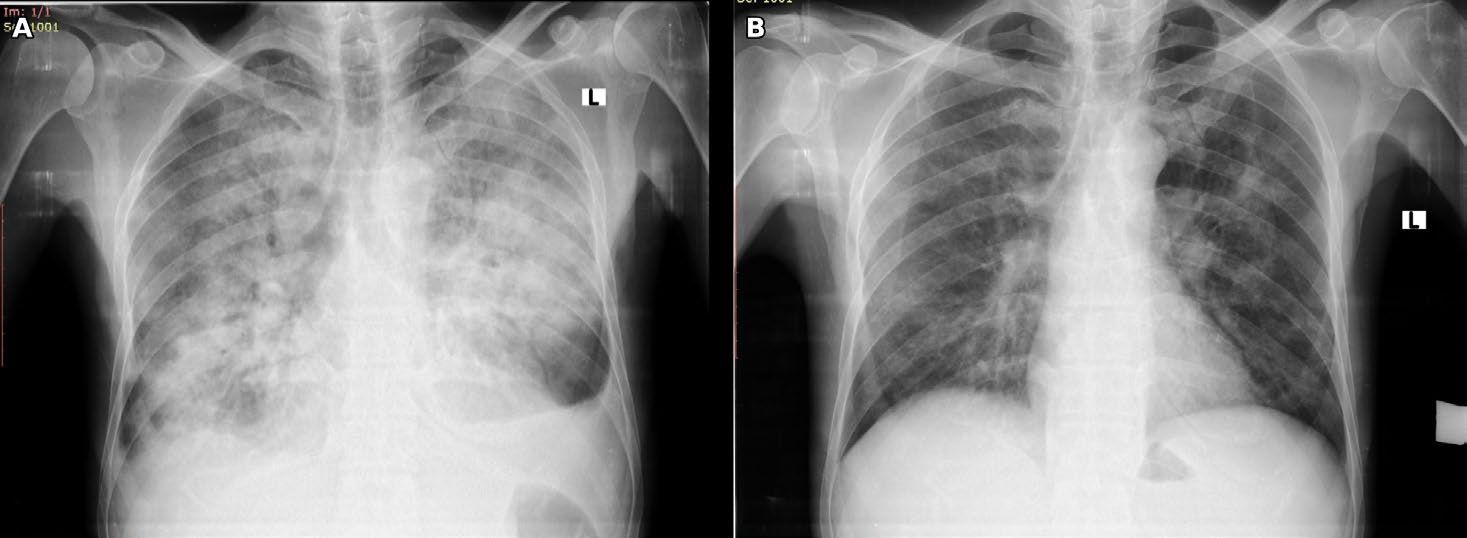
Chest radiography at admission (A) showing bilateral diffuse perihilar fluffy air‐space opacities with air bronchograms and blunting of left costophrenic angle, consistent with acute pulmonary edema, and at discharge (B) demonstrating near‐complete resolution of these findings following treatment.

White blood cell count had dropped to 21 × 10^3^/μL, hemoglobin had fallen to 7.8 g/dL, and platelet count had declined to 51 × 10^3^/μL. Most critically, the biochemical parameters demonstrated life‐threatening metabolic abnormalities consistent with TLS: serum creatinine had risen to 5.74 mg/dL, urea was markedly elevated at 209.5 mg/dL, serum potassium was dangerously elevated at 8.29 mmol/L, phosphorus was markedly elevated at 24.1 mg/dL, ionized calcium was critically low at 0.8 mmol/L, and uric acid had risen dramatically to 27.5 mg/dL. Serum LDH was also elevated to 368 U/L, reflecting massive cellular destruction (summary of key laboratory investigations are presented in Table 1). The electrocardiogram demonstrated peaked T waves and absent P waves, characteristic of severe hyperkalemia with cardiac toxicity. Chest radiography revealed bilateral diffuse air‐space opacities with prominent perihilar distribution, diffuse air bronchograms, and blunting of the left costophrenic angle consistent with bilateral pulmonary edema (Figure 1A). These radiographic findings, in conjunction with acute kidney injury, severe electrolyte abnormalities, and volume overload, supported TLS related pulmonary edema rather than primary cardiogenic or infectious causes of respiratory failure. All laboratory parameters are consistent with TLS‐induced metabolic derangement.

**TABLE 1:** Key laboratory parameters from diagnosis to discharge in a patient with chronic lymphocytic leukemia complicated by acute tumor lysis syndrome.

Laboratory parameter	Baseline (Day 0)	Day 3 (TLS presentation)	Day 7 (Postdialysis)	Day 14 (Discharge)	Reference range
Creatinine (mg/dL)	0.8	5.74	2.1	0.82	0.51–0.95
Potassium (mmol/L)	4.0	8.29	4.8	4.57	3.5–5.1
Uric Acid (mg/dL)	6.4	27.5	8.1	NA	2.6–6.0
Phosphate (mg/dL)	NA	24.1	5.6	NA	2.5–5.0
Ionized Calcium (mmol/L)	1.18	0.8	1.05	1.3	1.12–1.32
LDH (U/L)	167	368	215	217	120–300

*Note:* NA = not available.

**TABLE 2:** Peripheral blood flow cytometry findings and pathologic correlation.

Marker	Result (%)	Interpretation
CD45	99	Pan‐leukocyte marker; positive
CD19	98	B‐cell lineage marker; positive
CD5	99	Aberrant co‐expression on B cells
CD5/CD19	97	Dual positivity consistent with CLL/SLL
CD20	76	Moderate B‐cell marker expression
CD23	93	Positive; supportive of CLL/SLL
CD200	94	Positive; characteristic of CLL
CD10	0	Negative
FMC7	0	Negative
CD38	0	Negative
CD11c	22	Partial expression
CD25	0	Negative
CD103	0	Negative
Surface IgM	54	Positive
Kappa light chain	1	Negative
Lambda light chain	49	Positive; indicates light‐chain restriction

*Note:* Axillary lymph node biopsy revealed a malignant low‐grade non‐Hodgkin lymphoma consistent with small lymphocytic lymphoma (SLL).

### 3.2 Day 3–7: Intensive Management and Hemodialysis

Immediate resuscitative management was initiated. The patient received intravenous calcium gluconate to stabilize the myocardium against the effects of severe hyperkalemia. Potassium‐shifting therapy was instituted with intravenous insulin with dextrose as well as beta‐2 agonist. Aggressive diuresis with intravenous loop diuretics was initiated. Allopurinol (adjusted for renal impairment) was started immediately. Rasburicase, the preferred urate‐oxidase therapy with immediate efficacy, was unavailable in our resource‐constrained setting. Despite intensive medical management, the patient developed progressive refractory hyperkalemia potassium fluctuating between 7.5–8.2 mmol/L despite repeated interventions and severe fluid overload unresponsive to diuretic therapy, necessitating urgent hemodialysis. It was performed urgently in three sessions over the subsequent 48–72 h (Days 3–5 of hospitalization).

## 4 Conclusion and Results (Outcome and Follow‐Up)

### 4.1 Days 5–14: Recovery and Discharge

**FIGURE 2:**
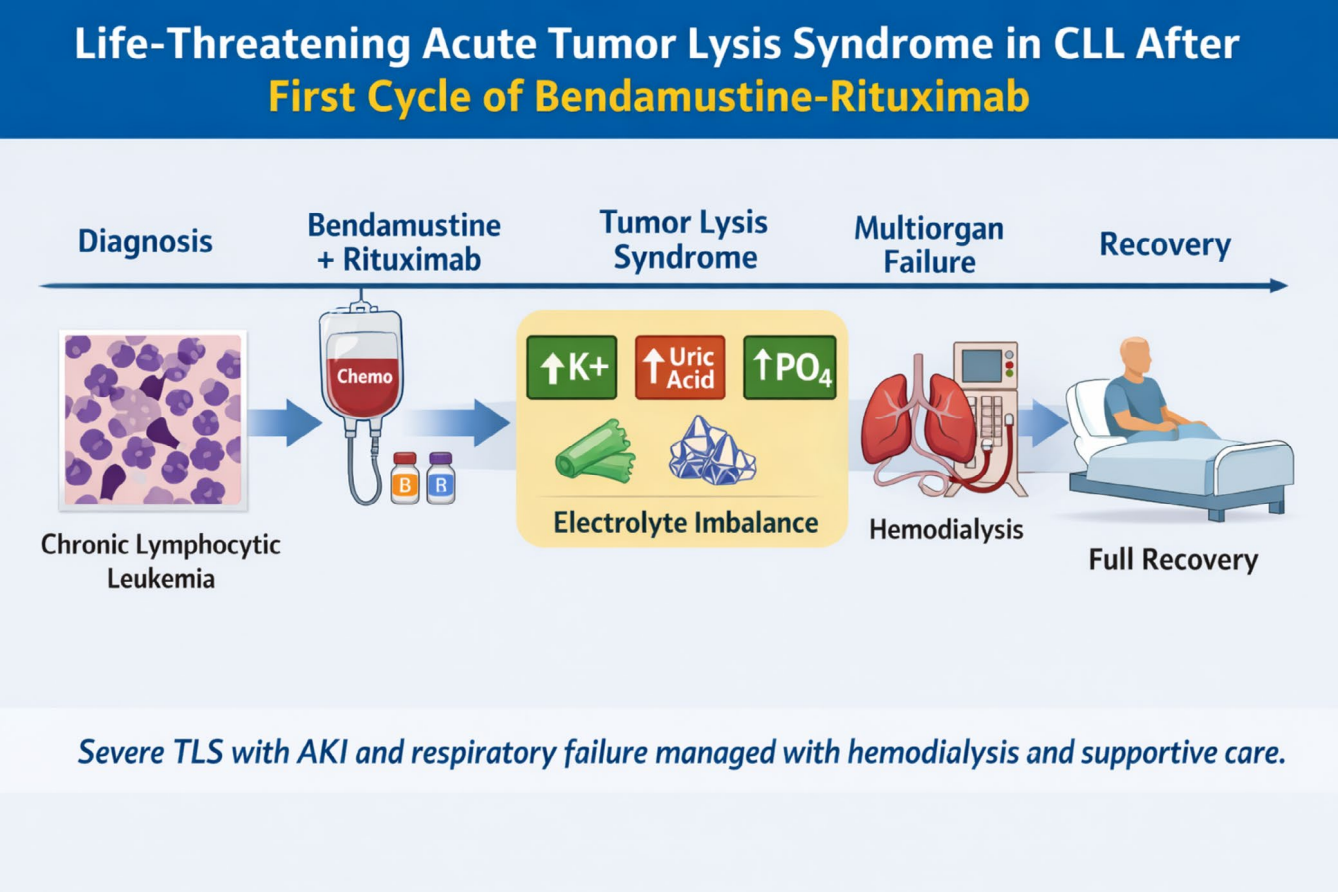
Graphical timeline of the patient's clinical course. The timeline illustrates key events in a patient with CLL who developed acute tumor lysis syndrome after the first cycle of bendamustine–rituximab, including TLS onset, multiorgan failure, hemodialysis, and full recovery.

Following hemodialysis, the patient's clinical condition and laboratory parameters improved markedly. Potassium levels normalized by Day 7, and serum creatinine gradually decreased, reaching 0.82 mg/dL by Day 14. Uric acid, phosphorus, and calcium levels also significantly improved. Oxygen requirements decreased, with complete weaning by Day 8. Urine output improved. The patient was transferred to a regular ward by Day 9 and discharged on Day 14 in stable condition, with normalization of renal function and near resolution of pulmonary edema on repeat chest radiography (Figure 1B). The timeline of key events is illustrated in Figure 2.

## 5 Discussion

### 5.1 Pathophysiology of Rapid‐Onset TLS in Advanced CLL

Acute TLS represents a medical emergency resulting from the catastrophic metabolic consequences of rapid malignant cell destruction [2]. The mechanism by which bendamustine–rituximab precipitates TLS in CLL is multifactorial and reflects the particular potency of this regimen. Bendamustine functions as a nucleoside analog and alkylating agent with strong immunomodulatory properties, inducing rapid apoptosis of malignant B lymphocytes. When combined with rituximab, a chimeric monoclonal antibody targeting CD20, the cytoreductive effect is substantially amplified through antibody‐dependent cellular cytotoxicity and direct complement‐dependent cytotoxicity [1, 3]. In patients with extreme tumor burden, this rapid cell death releases massive quantities of intracellular potassium, phosphorus, nucleic acids, and uric acid precursors into the systemic circulation [2].

The conventional paradigm that CLL represents a uniformly low‐risk disease for TLS reflects historical experience with typically indolent presentations and the relatively slow rate of spontaneous cell turnover in standard CLL [1, 2]. However, this generalization fails to account for the substantial subset of CLL patients who present with advanced disease features characterized by extreme leukocytosis, massive lymphadenopathy, and pronounced organomegaly [1]. The present case exemplifies this critical distinction: the patient's presentation with WBC of 300,000/μL and splenomegaly extending 20 cm below the costal margin represents extreme disease burden equivalent to lymphoma range disease in terms of tumor cell mass. The laboratory parameters and clinical features of the patients suggest high cellular turnover even before chemotherapy initiation. These features collectively define a high‐risk TLS population even within the CLL disease spectrum [2, 6].

### 5.2 Risk Stratification and the Failure of Traditional Classification

Contemporary risk stratification for TLS classifies patients into low, intermediate, and high‐risk categories based on malignancy type, tumor burden, and baseline biochemical parameters. Within this framework, CLL is generally categorized as a low‐risk disease, whereas aggressive lymphomas and acute leukemias are considered high risk [2, 6, 7]. However, emerging evidence demonstrates that this classification fails to capture clinically significant heterogeneity within CLL, particularly in patients with high tumor burden or when exposed to potent targeted or cytotoxic therapies [1, 7].

This misclassification represents a significant yet not uncommon clinical error, particularly in resource‐limited settings, and reflects the persistence of outdated paradigms in risk assessment. The case underscores that risk stratification for TLS must be individualized and should explicitly incorporate disease‐specific and patient‐specific factors rather than relying solely on traditional diagnostic categories [6, 8]. Importantly, contemporary evidence emphasizes that tumor burden is a principal determinant of TLS risk across malignancies, irrespective of whether it occurs in CLL, lymphoma, or other hematologic cancers [2]. Accordingly, patients with high tumor burden should consistently receive intensive prophylactic measures and close biochemical monitoring to mitigate life‐threatening complications. Advanced imaging may help in precisely quantifying tumor burden to risk‐stratify these patients [6, 8, 9].

### 5.3 Prophylaxis Strategies and the Resource‐Limitation Paradox

The standard approach to TLS prevention includes aggressive intravenous hydration to enhance renal perfusion and promote urinary excretion of potassium and phosphate, alongside urate‐lowering therapy and close electrolyte monitoring [2, 8]. Rasburicase, a recombinant urate oxidase, is the preferred agent in high‐risk patients due to its rapid conversion of uric acid into allantoin, a highly soluble metabolite efficiently cleared by the kidneys, thereby preventing nephrotoxicity. Multiple studies and guidelines have demonstrated that rasburicase is superior to Allopurinol in preventing clinical TLS and its complications [6, 8].

In resource‐limited settings, however, rasburicase is often unavailable due to cost and supply constraints, a challenge common in many developing‐world healthcare systems [2, 4]. As a result, allopurinol, a xanthine oxidase inhibitor with slower onset and inability to reduce pre‐existing hyperuricemia, frequently serves as the default prophylactic agent [2].

In the present case, the critical gap was not merely the absence of rasburicase but the failure to initiate even allopurinol prophylaxis prior to chemotherapy. This omission reflects both a misclassification of TLS risk and adherence to the outdated paradigm of CLL as universally low risk [1, 2]. Early initiation of allopurinol could have attenuated peak uric acid levels, potentially mitigating the severity of acute kidney injury and multiorgan dysfunction. This case underscores that, even in resource‐constrained settings, fundamental evidence‐based measures, including hydration and allopurinol, remain essential components of TLS prophylaxis.

### 5.4 Clinical Implications and Comparative Literature Context

Several prior reports have described bendamustine‐induced TLS in patients with CLL [4, 5]. The present case, however, exhibits distinctive features that enhance its clinical relevance. First, the rapid onset of TLS within 72 h following initial chemotherapy highlights the potent cytoreductive effect of bendamustine–rituximab in this patient population [3, 6]. Second, the severity of metabolic derangements necessitating urgent hemodialysis is uncommon in CLL‐related TLS and underscores the potential for catastrophic outcomes in high‐risk individuals [2, 8]. Third, the development of profound pulmonary edema requiring high oxygen support, while occasionally reported in TLS, reflects extreme fluid overload and capillary leak, which are not universal features [4, 10]. Finally, this case originates from a resource‐limited setting in sub‐Saharan Africa, where bendamustine–rituximab is increasingly used due to limited access to newer targeted therapies, yet awareness of TLS risk and prophylaxis may be comparatively lower than in developed healthcare systems [2, 4].

Emerging evidence also indicates that BTK inhibitors, such as ibrutinib and acalabrutinib, increasingly used as first‐line therapy in high‐income countries, can also precipitate TLS [11, 12]. This expands the population of CLL patients at risk and reinforces the necessity of individualized, universal TLS risk assessment irrespective of the therapeutic agent employed.

### 5.5 Prevention Strategies and Recommendations for Practice

The present case underscores several critical lessons for clinical practice. First, TLS risk stratification must be individualized rather than based solely on conventional disease categories. Patients with CLL presenting with marked leukocytosis (WBC > 100,000/μL), massive organomegaly, elevated LDH, or raised baseline uric acid should be considered high risk, irrespective of traditional disease classification [1, 2]. Second, all high‐risk patients should receive prophylactic urate‐lowering therapy prior to chemotherapy. While rasburicase is preferred when available, allopurinol at standard dosing represents an effective, lower‐cost alternative [6, 8]. Third, aggressive hydration and careful monitoring of electrolytes and renal function, initiated before and intensified immediately after chemotherapy, are essential [2, 10]. Fourth, patient education regarding warning symptoms including dark urine, oliguria, muscle cramps, palpitations, dyspnea, and neurological changes must be emphasized, with instructions for prompt medical evaluation if these occur [8]. Fifth, healthcare systems in resource‐limited settings must ensure access to hemodialysis, as TLS can progress rapidly to life‐threatening complications despite optimal medical management and may require urgent dialytic support [2, 4].

## 6 Patient Perspective

I was terrified when diagnosed with leukemia, but the doctors' explanations gave me confidence to start treatment. After chemotherapy, I became critically ill, with severe breathing difficulty and kidney failure. Prompt intervention, including dialysis, saved my life. I am grateful for the care provided, though clearer warnings about these severe symptoms would have been helpful.

## Author Contributions

**Malegna Temesgen Garuma:** conceptualization, data curation, formal analysis, methodology, project administration, resources, supervision, validation, writing – original draft, writing – review and editing. **Kebede H. Begna:** conceptualization, data curation, formal analysis, writing – review and editing. **Tesfaye Belete Amare:** conceptualization, data curation, formal analysis, investigation, methodology, resources, supervision, validation, writing – review and editing. **Mengisteab Kassahun Desta:** conceptualization, data curation, formal analysis, investigation, methodology, resources, validation, visualization, writing – review and editing. **Derebe Shashigo Leilago:** data curation, formal analysis, investigation, methodology, resources, validation, visualization. **Humud Mohammed Ahmed:** data curation, formal analysis, investigation, methodology, resources, validation, visualization.

## Funding

The authors have nothing to report.

## Ethics Statement

Not required for single‐patient case reports according to institutional policy.

## Consent

Written informed consent was obtained from the patient for publication of this case report and any accompanying images. A copy of the written consent is available for review by the Editor‐in‐Chief of this journal.

## Conflicts of Interest

The authors declare no conflicts of interest.

## Data Availability

All relevant data are included in this article. Additional information is available from the corresponding author upon reasonable request.
